# Whole-field visual motion drives swimming in larval zebrafish via a stochastic process

**DOI:** 10.1242/jeb.118299

**Published:** 2015-05

**Authors:** Ruben Portugues, Martin Haesemeyer, Mirella L. Blum, Florian Engert

**Affiliations:** 1 Department of Molecular and Cellular Biology, Harvard University, 16 Divinity Avenue, Cambridge, MA 02138, USA; 2 Max Planck Institute of Neurobiology, Sensorimotor Control Research Group, Martinsried 82152, Germany

**Keywords:** Locomotion initiation, Optomotor response, Zebrafish

## Abstract

Caudo-rostral whole-field visual motion elicits forward locomotion in many organisms, including larval zebrafish. Here, we investigate the dependence on the latency to initiate this forward swimming as a function of the speed of the visual motion. We show that latency is highly dependent on speed for slow speeds (<10 mm s^−1^) and then plateaus for higher values. Typical latencies are >1.5 s, which is much longer than neuronal transduction processes. What mechanisms underlie these long latencies? We propose two alternative, biologically inspired models that could account for this latency to initiate swimming: an integrate and fire model, which is history dependent, and a stochastic Poisson model, which has no history dependence. We use these models to predict the behavior of larvae when presented with whole-field motion of varying speed and find that the stochastic process shows better agreement with the experimental data. Finally, we discuss possible neuronal implementations of these models.

## Abstract (precis)

**Summary**: Initiation of swimming is based on an instantaneous evaluation of whole-field visual sensory input as opposed to an accumulation of evidence over a prolonged time.

## INTRODUCTION

When presented with whole-field visual motion, larval zebrafish will turn and locomote in the direction of perceived motion (Orger et al., 2008; Portugues and Engert, 2009). This innate reflex, called the optomotor response (OMR), is widespread in the animal kingdom and has been studied in a variety of animals. In insects, where the OMR has been most widely studied, it has been used to investigate, amongst other questions, the specifics of the elementary motion detectors (Buchner, 1976; Srinivasan et al., 1999; Borst, 2014), the differential tracking of translational and rotational whole-field motion (Zanker and Collett, 1985; Junger and Dahmen, 1991) and the visual control of flying speed (David, 1979; Baird et al., 2006; Fry et al., 2009). In zebrafish, the OMR has been used to assay visual acuity and identify visual system mutants (Neuhauss et al., 1999; Orger et al., 2003). In terms of its ethological importance, it is generally believed that the OMR ensures that animals remain in the same place with respect to their visual environment, at least for a range of behaviorally relevant speeds (Reichardt and Poggio, 1976; Severi et al., 2014).

Compared with other well-described innate reflexes like the vestibular ocular (Khater et al., 1993), eye blink (Disterhoft et al., 1977) or optokinetic reflex (OKR) (Dieringer and Precht, 1982), we show that the head-embedded forward OMR displays a remarkably long latency (>1.5 s) between stimulus onset and response. There are distinctly different models of neural dynamics that could cause such specific delays, and the mechanics of such models are of general interest in the field of neural coding and computation. Here, we show that the latency to initiate swimming depends strongly on the forward speed of the whole-field visual stimulus, which, in our case, is a square wave grating. What is the reason for this modulation in latency?

In order to understand which neuronal mechanisms give rise to the latency, we investigated whether the initiation of swimming in the context of the OMR is likely to be a history dependent or an instantaneous phenomenon. We present and test two biologically inspired models, which have been often used in neuroscience to understand single-neuron or network properties.

The first model is an ‘integrate and fire’ model. In this model the visual stimulus results in the accumulation of activity in an integrator. When the activity reaches a given threshold, the integrator fires a command signal and swimming is initiated. The second model is a ‘stochastic’ model. We assume that the initiation of locomotion is a Poisson process with a given rate, and that this rate can be modulated by various factors. In our specific case, the main factor is the visual stimulus that is presented to the larva. This means that if the stimulus being presented to the larva at time *t* is *s*(*t*), the probability *p*(*t*) of it evoking a swim in the larva in a small interval of time around *t* (the probability density function) is *p*(*t*)=*p*(*s*(*t*)), i.e. the probability is a function of the instantaneous stimulus. Therefore, if the stimulus changes over time, so will the probability (and rate) of the stochastic model. Importantly, these two simple models allow us to discriminate whether the initiation of swimming in the context of the OMR is likely to be a memory-dependent or rather an instantaneous process.

These models have a rich history in neuroscience and have been used extensively to study the firing properties of neurons and how these may relate to activity observed on a network level. In particular, they have been used to address the question of how observed stochastic cortical activity (Softky and Koch, 1993) can be reconciled with the rather deterministic firing properties of individual neurons (Shadlen and Newsome, 1998; Stevens and Zador, 1998). The suggested mechanisms – balance of excitation and inhibition (Shadlen and Newsome, 1998; van Vreeswijk and Sompolinsky, 1996) or influence of short bursts of activity (Stevens and Zador, 1998) – have constrained many future studies of neuronal computation.

Here, we use the integrate and fire model and the stochastic Poisson model in a behavioral rather than neuronal setting to describe the initiation of behavior as opposed to the generation of a spike. Our aim is to provide constraints on the neuronal mechanisms that could be involved in initiation of locomotion.

**List of symbols and abbreviations**

*a*: acceleration
IG: inverse Gaussian distribution
*L*: latency
OKR: optokinetic reflex
OMR: optomotor response
*T*_th_: total activity threshold
*v*: speed

## RESULTS

### Latency to initiate swimming as a function of grating speed

**Fig. 1.**
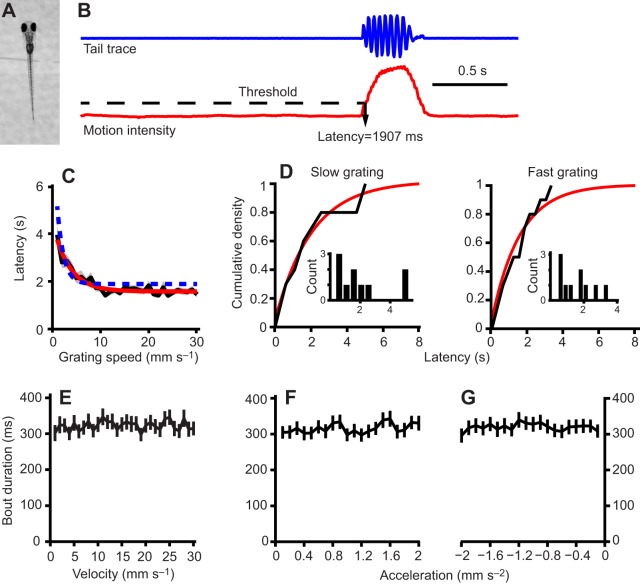
**Latency to initiate swimming** **in larval zebrafish** **depends on the speed of whole-field motion.** (A) Head-restrained larval zebrafish with its tail free to move. (B) The tail is tracked and the cumulative angle of all ten body segments is computed in real time (top trace). The start of bouts can be detected automatically when tail motion intensity (bottom trace) exceeds a threshold. This particular trial corresponds to a trial of speed 11 mm s^−1^ for one example fish. (C) The latency as a function of forward grating speed averaged over 102 larvae (black trace, s.e.m. in gray). The fit of the form α+β*e*^−γ*v*^ is shown for the average data (red curve, α=1.579, β=2.922 and γ=0.296) and for the same example fish as in B (blue curve, α=1.892, β=6.921 and γ=0.754). (D) Distribution of latencies for trials in two speed bins: grating speeds from 6 to 10 mm s^−1^ (left) and from 16 to 20 mm s^−1^ (right) for the same sample fish as C. The insets show the histograms of counts and the main graphs in black show the cumulative density. In red are the cumulative probability functions for an exponential distribution with mean equal to the mean of the latencies in that bin. (E) Average bout duration across all fish as a function of the speed of the grating that was presented and triggered their initiation. (F,G) Average bout durations for the experiments in which gratings accelerated from rest at a constant acceleration (F) and decelerated from 10 mm s^−2^ with a constant deceleration (G). The bout duration is plotted as a function of the acceleration.

Zebrafish larvae were head restrained as described in the Materials and methods (Fig. 1A). A trial began when the grating started moving in the caudo-rostral direction at a fixed speed between 1 and 30 mm s^−1^. The grating stopped moving as soon as the larva started swimming, although data acquisition was terminated 1 s after the fish started swimming (Fig. 1B). The inter-trial interval was 30 s. All integer speeds between 1 and 30 mm s^−1^ were tested twice in a randomized fashion, resulting in 60 total trials per fish (*N*=112 fish total). The results for this latency *L*(*v*) (measured in seconds) as a function of grating speed *v* are shown in Fig. 1C. For each fish, we fit the dependence of its individual latency on grating speed with a curve of the form:
(1)
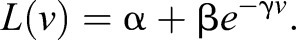

One such fit is shown in Fig. 1C as a blue line. This functional form was chosen because it both captures the most salient features of the behavior, namely a decay for small speeds to an asymptotic value, and it allows an analytical solution to be found for certain models to be considered later. The average population fit was evaluated by fitting the parameters α, β and γ to the fish average latencies. The population fit was found to be:
(2)
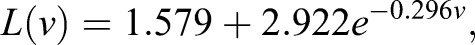


which is shown as a red line in Fig. 1C. A qualitatively similar dependence of initiation latency on stimulus speed has been observed in the OKR of frogs (Dieringer and Precht, 1982). Even though the average response latencies in the case of the OKR are considerably lower, it does suggest that this type of speed dependence may be shared across different visual behaviors.

It is interesting to note that although the latency to initiate swimming depended on the speed of the grating, the duration of the bout did not (Fig. 1E–G). As we know that bout parameters depend on grating speed (Severi et al., 2014), this may be attributed to the fact that in our experiments, the grating came to rest as soon as the bout was initiated. This indicates that bouts are not performed in a ballistic fashion and that fish dynamically update their swimming based on changes in grating speed within a bout as has been previously reported by Portugues and Engert (2011).

### Distribution of latencies

Apart from the dependency of average latencies on grating speed, we wished to gain some insight into the distribution of these latencies in order to understand their neuronal underpinning. As each constant speed was only tested twice, we did not have enough trials to address this question. Therefore, we binned trials for each fish into six bins depending on their speed: bin 1 included trials with speeds between 1 and 5 mm s^−1^, bin 2 included trials with speeds between 6 and 10 mm s^−1^ and so on up to bin 6, which included trials with speeds between 26 and 30 mm s^−1^. Thus each bin contained ten trials.

We then asked for each larva whether the distribution of latencies in each bin differed significantly from that expected from an exponential distribution with the same mean (Fig. 1D). Using a Kolmogorov–Smirnov test we were able to reject the null hypothesis (*P*<0.05) in only 72 of the 612 bins. In fact, correcting for multiple comparisons using a Bonferroni correction led us to reject the null hypothesis in only 16 out of the 612 cases. We therefore interpreted this as an indication that the distribution of latencies we observed could have arisen from an exponential distribution underlying a stochastic model. We do note, however, that given the small sample size and noise inherent in biological systems this observation cannot rule out an underlying history-dependent process.

### Modeling the distribution of latencies

**Fig. 2.**
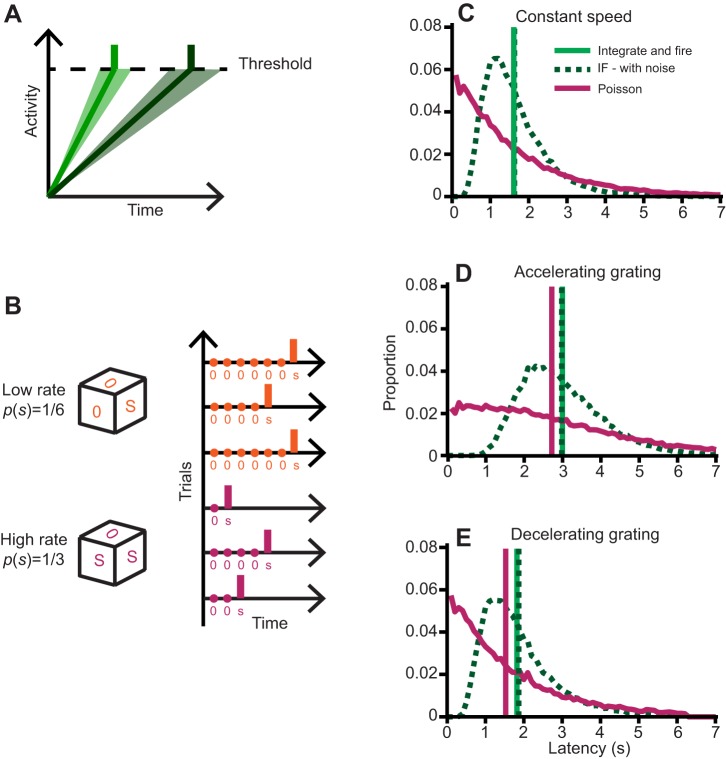
**Example distributions of modeled latencies.** (A) Illustration of the integrate and fire model, showing history dependence. The light green line symbolizes a high rate or fast accumulation (steep gradient) resulting in a short latency and the dark green line a low rate (shallow gradient) leading to a longer latency. Shaded areas represent variance for our integrate and fire (IF) model with noise. (B) Illustration of the Poisson model. The model can be represented by rolling dice at every instant in time and thus having no history dependence. The average response latency across trials is then determined by the probability of success *p*(*s*) on a given trial (low rate trials orange, high rate trials magenta). (C) Distribution of modeled latencies for the integrate and fire model, the integrate and fire model with noise and the Poisson process at constant velocity *v*=15 mm s^−1^. Mean latencies, indicated by vertical lines, are 1.61 s for the integrate and fire model, 1.63 s for IF with noise and 1.60 s for the Poisson model. (D) Distribution of modeled latencies for accelerating gratings. Initial grating speed was *v*_0_=0 mm s^−1^ and the acceleration was *a*=1.6 mm s^−2^. Mean latencies are 3.02 s for the integrate and fire model, 2.98 s for IF with noise and 2.72 s for the Poisson model. (E) Distribution of modeled latencies for decelerating gratings. Initial grating speed was *v*_0_=10 mm s^−1^ and the acceleration was *a*=−1.6 mm s^−2^. Mean latencies are 1.82 s for the integrate and fire model, 1.87 s for IF with noise and 1.53 s for the Poisson model.

The aim of our study was to investigate whether these observed latencies are the result of a history-dependent process that accumulates sensory evidence over time or whether they arise as the output of a stochastic model whose rate is set by the instantaneous sensory input being perceived and is therefore a history-less process. Processes that involve the accumulation of sensory evidence require three main steps. The first is the translation of sensory input into a rate or activity. Subsequently, this activity has to be integrated over time. Finally, a criterion needs to be implemented: when does the accumulation of evidence trigger a decision, which in our case would result in the initiation of locomotion. The most common criterion to implement is that of a threshold. Threshold models are rather general and could encompass various *a priori* plausible strategies. For example, we could imagine fish starting to respond a given fixed amount of time *T* after noticing whole-field motion. This would correspond to a rate *r*=1/*T* independent of the velocity. By contrast, a different strategy would have fish initiate swimming after the visual scene had moved forward a given distance *D* (a fixed amount of optic flow). This would correspond to a rate *r*(*v*)=*v*/*D*. The rate we observe (given in Eqn 7 in the Materials and methods, which is deduced from Fig. 1C) is inconsistent with either of these two simple strategies. We therefore propose a model in which the whole-field motion results in a rate that is a function of the velocity and where this rate is integrated over time until a threshold is reached, upon which swimming is initiated (Fig. 2A). We call this an ‘integrate and fire’ model.

We based our stochastic model on a Poisson process. A Poisson process is memory-less by construction, with average response latencies depending on the instantaneous response probability alone. A Poisson model therefore allows us to contrast a history-independent process with evidence accumulation represented by an integrate and fire model. Like threshold models, a Poisson model could encompass various *a priori* plausible strategies through its simple relationship of average response latency *L* to rate λ (see Materials and methods). For the same reasoning as presented above, we construct a model in which the rate of a Poisson process is a function of grating velocity. However, instead of the rate setting the accumulation in an integrator, it directly influences the response probability *p*(*s*) and hence the average latency to respond (Fig. 2B).

To that end, we first fitted both models to the data summarized in Eqn 2 and then tested their predictive power with experiments described below.

### Latency to initiate swimming when presented with a grating of varying speed

For gratings moving at constant speed, both the integrate and fire (Fig. 2A) and the Poisson model (Fig. 2B) will, by construction, predict the same average latency to initiate swimming (Fig. 2C). The integrate and fire model presented above is purely deterministic: it will produce the exact same outcome every time. We expect biological models to be noisy and exhibit variance, and our data indeed does so. We therefore also considered the introduction of noise into the integrate and fire model (see Materials and methods). Fig. 2C shows that the addition of noise to the integrate and fire model has the effect of turning a purely deterministic response into a wider distribution of response latencies without affecting the mean latency.

**Fig. 3.**
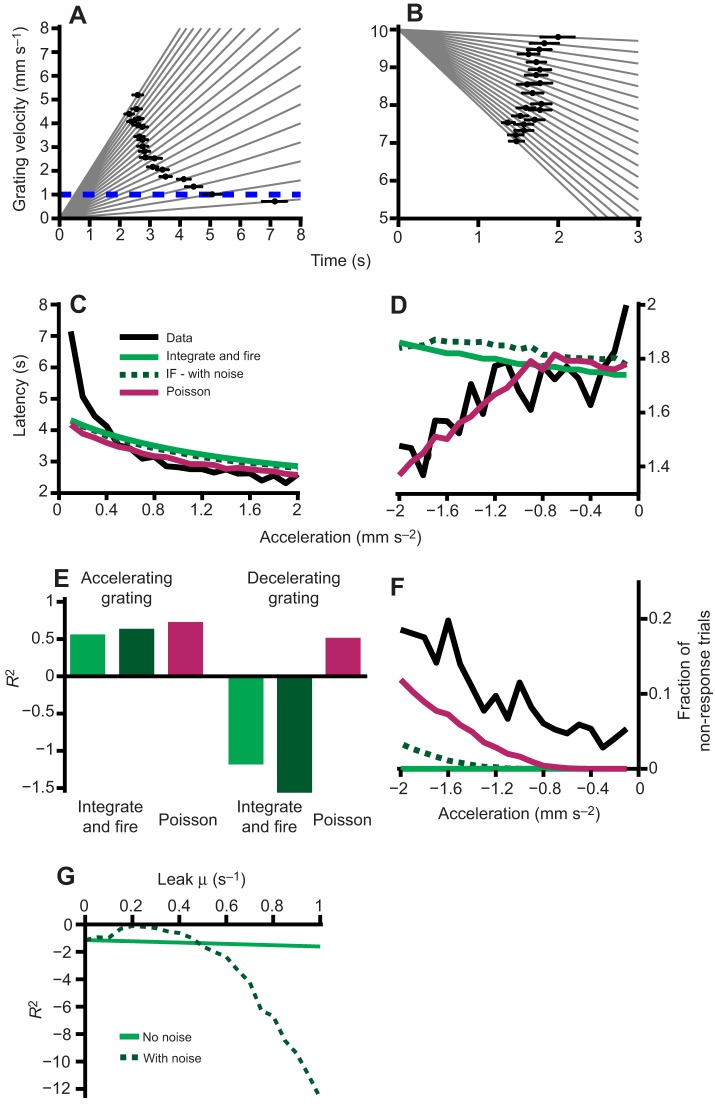
**A Poisson model better predicts experimental data.** (A,B) Observed latencies to respond when fish are presented with an accelerating grating or decelerating grating, respectively (black circles±s.e.m.), overlaid onto the grating speed, which is shown in gray lines for each acceleration/deceleration. The dashed blue line in A indicates the minimum speed covered by our velocity fit. (C,D) Experimental latencies to respond (black line) and fits for the integrate and fire model (light green), integrate and fire with noise (dotted dark green) and the Poisson model (magenta) versus acceleration or deceleration of the grating, respectively. (E) Goodness of fit between the models and the actual data, for accelerating gratings (left; *R*^2^=0.56 integrate and fire model; *R*^2^=0.64 integrate and fire with noise; *R*^2^=0.73 Poisson model) and decelerating gratings (right; *R*^2^=−1.22 integrate and fire model; *R*^2^=−1.61 integrate and fire with noise; *R*^2^=0.53 Poisson model). (F) Fraction of trials without response before the grating came to rest in decelerating trials for the experimental data (black line) and the models. (G) Effect of a leak term μ on the goodness of fit of integrate and fire models. The solid light green curve indicates the *R*^2^ for a leaky integrator model while the dotted dark green curve is computed for a leaky integrator with noise (Best fit: *R*^2^=−0.12 with μ=2×10^−4^).

We next sought to devise a set of experiments that would allow us to test which model better predicts the distributions of latencies. As shown in Fig. 2D,E, when presented with either an accelerating or decelerating grating, both the deterministic and the noisy integrate and fire model predict a different average latency than the Poisson model. Therefore, we presented fish with either an accelerating or decelerating grating, according to the linear formula:
(3)
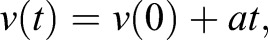


where *v*(0) is the initial velocity of the grating and *a* is the acceleration, which may be positive or negative. For the accelerating grating experiment, *v*(0)=0 and *a*=0.1, 0.2, …, 2 and for the decelerating grating experiment *v*(0)=10 and *a*=−0.1, −0.2, …, −2. Just as before, we measured how long it takes for the larvae to initiate swimming as a function of the acceleration. Fig. 3A shows for each positive acceleration the mean latency to respond overlaid on the grating speed which linearly increases with time. Note that as the acceleration increases, the latency to respond decreases, which is expected based on the dependence of response latency to grating speed (Fig. 1C). Importantly, the responses occur neither at a fixed time after grating start nor at a fixed velocity. Fig. 3B shows the equivalent data for decelerating gratings. For these experiments the relationship between acceleration and latency is more complex as fish occasionally fail to respond (see Fig. 3F) before the grating speed reaches *v*=0, at which time we stop the experiment. This results in an apparent decrease of mean response latency for more negative accelerations because failures to respond do not contribute to the mean latency.

### Comparing the goodness of fit

Fig. 3C depicts the observed latencies for accelerating grating as well as the predicted latencies for the integrate and fire model (with and without noise) and the Poisson model. We note (also see Fig. 3A) that for the slowest acceleration (*a*=0.1 mm s^−2^) experimental fish respond while the grating speed is smaller than 1 mm s^−1^. Since we did not test any constant speeds below 1 mm s^−1^, our fit in Eqn 1 is not able to predict the latency well for these speeds. We therefore do not expect our models to fit the experimental data well for the lowest presented accelerations. Fig. 3D compares observed and predicted latencies for the decelerating gratings. Qualitatively, both figures show a better fit of the Poisson model to the experimental data.

In order to quantitatively compare the models and determine which one fits the observed experimental data better, we evaluate their goodness of fit by determining the sum of the square residuals, that is, the sum of the squares of the difference between the data and the model fit. We determined the latencies of our integrate and fire model without noise and ran 10,000 simulations for the Poisson model and the Integrate and fire model with noise, averaged the results and computed the goodness of fit (see Materials and methods), which is shown in Fig. 3E. These indicate that the Poisson model provides a better fit to the data for both the accelerating and decelerating grating experiments than either of the integrate and fire models. In fact for the decelerating grating, both integrate and fire models have less predictive power than the mean of the data. For the accelerating grating, the integrate and fire model with noise performs better than the purely deterministic model, but this is reversed for the decelerating grating experiment.

As mentioned above, a consequence of the decelerating grating is that fish will not always respond before the grating has come to rest. This is shown in Fig. 3F for the experimental data, as well as for the model predictions. Failures to respond are an important outcome of the decelerating grating experiment because they effectively remove the tail of the experimental and model response latency distributions, which results in a reduction of the apparent mean latency. While neither of our models fully predicts the fraction of failed responses observed in the experimental data, Fig. 3F shows that this feature is better approximated by the Poisson model than by the noisy integrate and fire model. The noiseless integrate and fire model is completely deterministic and therefore cannot result in a failure fraction that is different from either 0 or 1, depending on the starting speed of the simulation. It therefore cannot represent this feature of the data. The addition of a leak-term to the noisy integrate and fire model can improve the goodness of fit for the decelerating grating experiments (Fig. 3G). However, even for an optimized leak term of μ=2×10^−4^, the model is still worse than the data mean in predicting the experimental results with *R*^2^=−0.12. In summary, we note that the Poisson model is considerably better in predicting different features of the experimental data than the integrate and fire model (Fig. 3E,F).

## DISCUSSION

In this study we have shown that the latency to initiate locomotion when presented with a whole-field moving visual stimulus depends on the speed of the stimulus. This latency can vary by up to a factor of two, from ∼4 to ∼2 s. These relatively long and variable response times have also been observed in other species (see Hanes and Schall, 1996; Luce, 1986). What underlies this modulation?

One way in which latencies that extend over these timescales can arise is through the processing and integration of sensory drive. This requires the nervous system to accumulate and store this sensory evidence over several seconds before a ‘decision’ can be made. On the other hand, one may envision strategies by which behavior arises through purely local spatio-temporal rules that do not require any memory component. This could be brought about by a stochastic process whose rate is dependent on the sensory input at that particular instance in time. In this way, increased sensory drive just results in a greater chance of performing the behavior.

Both these strategies are implemented in the nervous system. Responses of single neurons are often modeled as deterministic and represented by integrate and fire models (Burkitt, 2006). On a systems level, for example, the activity of integrating neurons in the frontal eye field is believed to control saccadic eye movements (Schall and Thompson, 1999). More recently, modeling studies have been used to argue that neurons in the lateral geniculate nucleus are better described by integrate and fire models rather than Poisson models (Lin et al., 2012): this both reproduces their statistical firing properties better and confers higher direction selectivity to area V1 of the visual cortex. In addition, integrators can also be realized on a network level, as in the oculomotor integrator (Robinson, 1989). We expect any biological system to decay to its resting state in the absence of input. This is usually represented by a leak term in the integrator, which for single neurons would correspond to a decay constant in the membrane potential. Introducing such a term into our model does not change the conclusions we presented above (Fig. 3G).

Stochastic models have also been applied in neuroscience. It has been observed in cortical networks that inter-spike intervals are exponentially distributed (Softky and Koch, 1993), leading to the idea that this activity follows a Poisson process in which it is the spike rate that is of neuronal relevance (Shadlen and Newsome, 1994).

**Fig. 4.**
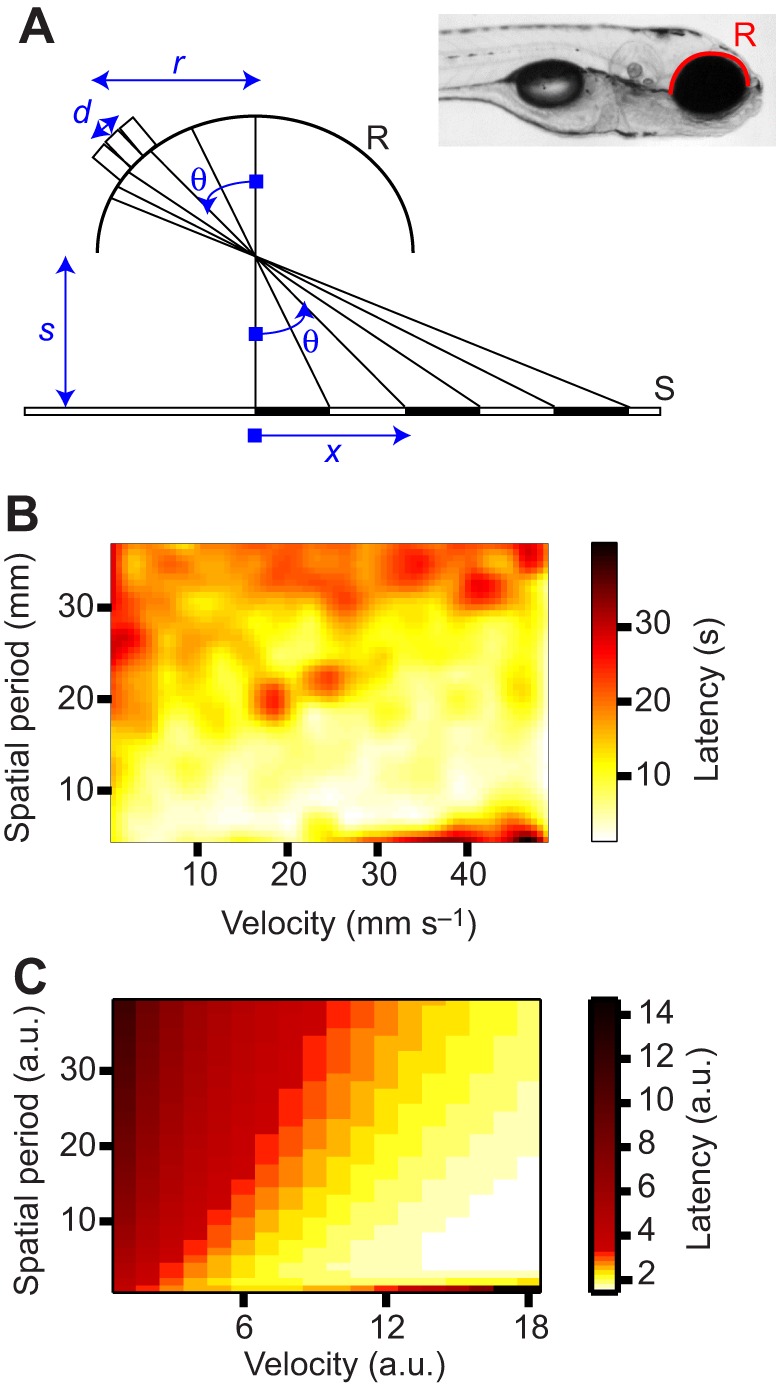
**The retina as a device that computes the rate of passing edges.** (A) Geometry of the model considered. A retina R of radius *r* consisting of a semi-circumference looks down on a screen S which is placed a distance *s* from its center (see inset illustrating the case in our experiments). A square wave grating of period *w* is shown on the screen. The coordinate *x* on the screen is related to the angular coordinate θ on the retina as mentioned in the text. (B) Latency to initiate swimming as a function of both grating velocity and spatial period of the grating. The plot shows the latency averaged over 26 larvae. (C) The activity from our model is either integrated by a non-leaky integrate and fire model or used in a Poisson model to result in a latency.

Both the integrate and fire model and the Poisson model require an input that is set by sensory drive. In our case, this sensory drive is related in some unknown way to the velocity *v* of the grating. These input variables are *r*(*v*) and λ(*v*) in Eqns 7 and 13, respectively. In our case, these could arise from an edge-counting mechanism in the retina (see Fig. 4 and technical derivation in the Appendix, for a simple model).

In our study, we find that a Poisson model fits the experimental data more accurately than an integrate and fire model. The noiseless integrate and fire model is purely history dependent, whereas the Poisson model is purely instantaneous: the rate is set at any moment in time uniquely by the stimulus that is being presented at that instant. The addition of noise, which by the above definition is always instantaneous, can be thought of as shifting the integrate and fire model from a history dependent one to a more instantaneous one (see Appendix). We do note, however, that the moderate addition of noise does not result in a considerably better fit to the observed data (see Fig. 3E). This suggests that zebrafish larvae use mainly instantaneous sensory information to initiate swimming. In fact, it has been observed that behaviors that extend in both space and time can arise from purely local rules, such as bacterial chemotaxis (Berg, 1993). Our experiments do not investigate the neural mechanisms that underlie this Poisson process. We do note, however, that head-restrained larval zebrafish like the ones in our experiments do perform spontaneous swims at a very low rate (∼0.01 Hz), which could be an indication of a locomotor network that stochastically crosses threshold. It could be that visual drive regulates this network through neuromodulation by dynamically decreasing the difference between its internal state and the threshold to initiate locomotion.

We do not know where in the nervous system this stochastic network may be. One possibility is that it could control the activation of command-like neurons akin to the Mauthner cell, which initiates escapes (Korn and Faber, 2005), or nuclei such as the nucleus of the medial longitudinal fasciculus (nMLF), which has been demonstrated to be involved in the control of swim speed in larval zebrafish (Severi et al., 2014). Command centers that can elicit locomotion upon stimulation have long been studied in other species such as the mesencephalic locomotor region in cats (Shik et al., 1969) or the tegmentum in teleost fish (Kashin et al., 1974). Alternatively, it could be implemented within the spinal cord itself, where sensory drive sets the difference between baseline and threshold network activity, in which case it would be stochasticity within the spinal network that elicits swimming (Buchanan and Grillner, 1987).

It has been observed across many animals and behaviors that latencies to response initiation are variable and often longer than can be explained purely by neuronal transduction delays (see Hanes and Schall, 1996; Luce, 1986). Here, we observe a similar phenomenon in larval zebrafish in the context of the optomotor response. Using a modeling approach, we propose that swim initiation is controlled by a stochastic network. We find this to be a rather elegant mechanism because it is an example of how local spatiotemporal rules can give rise to seemingly more complex behaviors.

## MATERIALS AND METHODS

### Preparation of head-restrained fish

Larval zebrafish at 6 to 8 days post fertilization were embedded in a 35 mm Petri dish in 2% agarose. After setting, the agarose around the tail was removed as described by Portugues and Engert (2011) (Fig. 1A). Larvae were shown a square-wave grating moving in a caudo-rostral direction. The period of the gratings was 10 mm and it was projected, with a 3M mobile projector, on a Nielstoff screen 5 mm below the embedded fish. The setup was illuminated with infrared LEDs and imaged from above with a Pike camera (AVT) at 200 Hz. Custom-written software in Labview (National Instruments) displayed the grating, and tracked the tail of the larva in real time. Tail motion was summarized by computing the cumulative tail angle and tail motion intensity was computed by calculating the standard deviation of the cumulative tail angle in a 50 ms time window (Fig. 1B). The initiation of a swim bout was detected automatically when tail motion intensity exceeded a manually determined threshold (the same for all fish). As soon as a bout was initiated, the grating was stopped and the trial was terminated, although data was acquired for 1 more second. No fish were discarded in the analysis.

### Experimental paradigm

The experiment consisted of 180 trials, 60 of which were with the grating moving at constant speed, 60 with the grating accelerating and the other 60 with the grating decelerating. The order of the trials was randomized. In the constant speed trials, the grating moved at a speed of 1 to 30 mm s^−1^ (in 1 mm s^−1^ steps). In the constant acceleration trials, the grating started from rest at the beginning of the trial and accelerated with a constant acceleration of 0.1 to 2 mm s^−2^ (in 0.1 mm s^−2^ steps). Finally, in the constant deceleration trials, the grating started moving at a speed of 10 mm s^−1^ and decelerated with a constant acceleration of −0.1 to −2 mm s^−2^ (in −0.1 mm s^−2^ steps).

### Latency distribution

As outlined in the Results, we tested whether the observed latencies could arise from an exponential distribution by binning latencies for five consecutive speeds (a total of 10 trials) and using a Kolmogorov–Smirnov test to test the null hypothesis that the observed latencies come from an exponential distribution with the same mean as the data mean. The total number of bins across all experiments was 612 (6 bins per fish, 102 fish total). The null hypothesis was rejected for *p*<α with α=0.05 or α=0.05/612 after Bonferroni correction for multiple comparison, respectively.

### Modeling

Numerical modeling was performed by using custom written software in MATLAB (MathWorks). Explicitly, integrate and fire models were modeled using the initial conditions:
(4)
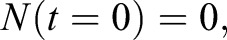
together with the evolution equation:
(5)
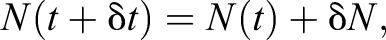


where δ*N* is given by Eqns 6, 8 or 9 for the pure integrate and fire, noisy integrate and fire and leaky integrate and fire models, respectively. The latency was defined as the first time point for which *N*≥1.

#### Integrate and fire model

Integrate and fire models have been extensively used to describe single neuron membrane potentials. In these models, voltage changes resulting from the opening of conductances integrate in time and result in a membrane potential which is history dependent. Furthermore, when the membrane potential reaches the threshold voltage for the particular neuron, it fires before resetting itself and returning to its resting value. Our model is closely analogous to this (Fig. 2A). Instead of representing the firing of a neuron, we aim to describe a different outcome: initiation of locomotion. The stimulus is perceived and processed and a signal is passed onto an integrator, which may be a brain region or a single neuron. When activity in the integrator reaches threshold, a command is sent that results in swimming and activity in the integrator is reset to its original value.

To explain our base experiment, the rate of increase in the activity of the integrator must be a function of grating speed, as this is the only parameter varied in the experiment. Explicitly, the assumptions that enter this model are: (1) swims are initiated when the activity *N*(*t*) in the integrator reaches threshold; (2) the activity threshold *N*_th_ that elicits swimming is constant and equal to 1; (3) the rate of increase in activity *r*(*v*) is a function of the instantaneous grating speed *v* only; and (4) the integrator can be a simple integrator or a leaky integrator.

The above assumptions imply that activity in the integrator changes according to the formula:
(6)
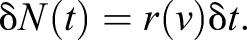


We define the latency *L*(*v*) as the time at which *N*(*t*) crosses threshold. We are considering models with a constant threshold and non-constant rates (Hanes and Schall, 1996), so without loss of generality, we set the threshold of the integrator of our model to 1. Therefore, the product of the rate and the latency is equal to 1, which implies, together with Eqn 1, that:
(7)
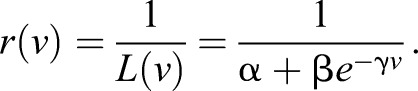


#### Integrate and fire with noise

The integrate and fire model presented above is purely deterministic: it will produce the exact same outcome every time. However, we expect biological systems to be noisy and exhibit variance and our data indeed does so. A natural way to do this is to add Gaussian noise to the above model
(8)
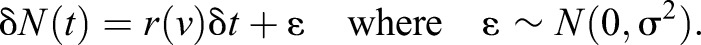


This model is analogous to the random walk models with drift (see Burkitt, 2006 and Tuckwell, 1988, where they are also referred to as Wiener processes) that were first introduced to simulate stochastic neuronal responses (Gerstein and Mandelbrot, 1964). These models have more recently been used to understand cortical neurons in the context of decision making tasks (Shadlen and Newsome, 1994, 1998). In terms of parameters, the rate *r*(*v*) in Eqn 8 corresponds exactly to the drift parameter of the random walk. The introduction of noise renders the integrate and fire model non-deterministic and allows a more natural comparison with the stochastic Poisson model introduced below (see Appendix for details).

#### The leaky integrator

The equation governing the change of activity in a leaky integrator model with leak rate μ is a modified version of Eqn 6 given by:
(9)
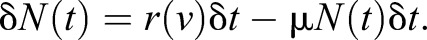


#### Poisson model

A Poisson process is a stochastic process which occurs at a constant rate in time determining the average latency of events. The probability of an occurrence in a small time period δ*t* is given by:
(10)
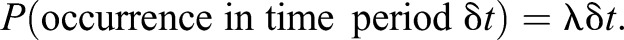


This process is memory-less in the sense that the probability of an occurrence during the time window δ*t* is completely independent of what has happened before (Fig. 2B). The time between these occurrences is described by an exponential distribution of latencies with parameter λ and probability density function:
(11)
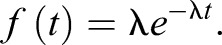


The mean 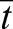 is equal to the variance σ:
(12)
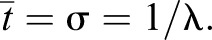


In our model, we consider swim initiation to be a stochastic process, with a rate imposed by the speed *v* of the grating. This implies that from Eqn 1:
(13)
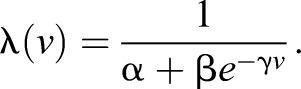


A consistency test that is often used to check whether a process could be Poisson or not is that its coefficient of variation (CV), that is, the ratio of the mean to the standard deviation of the measurements should be equal to 1. This indicator is not sufficient to prove this and instead as discussed above, we decided to test whether the latencies were consistent with an exponential distribution (Fig. 1D). We also note that in both our models, we could include sensory-relaying delays in the order of tens of milliseconds. For simplicity, given that we expect these delays to be much shorter than the timescales of the behavior, we decided to proceed without them.

When we allow *v* itself to be a function of *t*, according, for example, to the trajectory in Eqn 3, the process becomes what is known as an inhomogenous Poisson process (see Dayan and Abbott, 2001). The probability of a fish initiating a swim in a time interval (*t*, *t*+δ*t*) becomes λ(*t*)δ*t*, where the dependence of λ on *t* arises through the dependence of λ on a time varying velocity: λ(*v*(*t*)).

### Goodness of fit

The goodness of fit of the models was evaluated as the coefficient of determination. The total sum of squared distances for each model and data point was calculated as per:
(14)
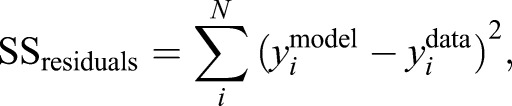
and the total sum of squared distances of each data point from the data mean was calculated as per:
(15)
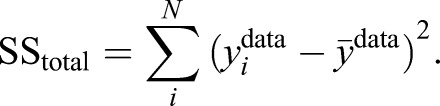


Using Eqns 14 and 15, *R*^2^ was calculated as:
(16)
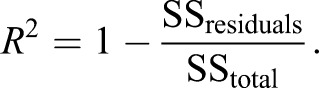


Therefore *R*^2^ will be equal to 1 if the prediction of the model is exact and smaller than 0 if the model is worse in predicting the data than the data mean. We note that since we predict the data using our models, rather than fit the data using for example linear regression, *R*^2^ values are not bounded below by −1.

## Figures and Tables

**Fig. A1.**
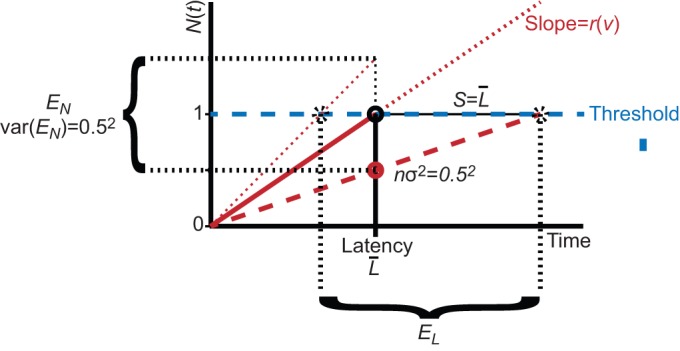
**Noise estimation for the integrate and fire model.** The relationship between noise in the integrator value (*E_N_*) and resulting noise in the latency (*E_L_*). As shown in the figure, per Eqn 6 for our threshold value of 1 (dashed blue line) the mean value *N*(*t*) of the integrator rises with slope *r*(*t*)=1/*L* as depicted by the solid red line, in order to reach an average latency *L*, depicted by the solid black line and circle. To result in a threshold crossing at the time *T*=*L*+*S* with *S*=*L*, the slope of our integrator has to be *r*(*v*)/2, shown by the dashed red line. In that case, at time *L*, our integrator value *N*(*t*) would be equal to 0.5 as shown by the solid red circle. Therefore, we set the s.d. of our total error *E_N_* in the integrator to 0.5. This does result in an error *E_L_* in the latency that has an s.d. *S*<*L*, which is distributed asymmetrically around the latency *L*, as visualized by the location of the stars.

## APPENDIX

### Adding noise to the integrator model

While our model based on a Poisson process (see the Materials and methods) inherently generates stochastic responses, the latency of an integrator model is deterministic. Our experimental data suggest that response latencies are variable. We therefore sought to introduce noise into our integrate and fire model that would partially match the variable nature of the latencies predicted by the Poisson model.

#### Non-leaky integrator

Since we do not know the actual source of the noise in our system, we assume the simplest model, Gaussian noise that is added at each time step as follows:

(A 1)

We note that this introduction of noise into the integrate and fire model is akin to a Wiener process *W*(*t*) with drift where our rate *r*(*v*) sets the drift while the noise scales the random walk of the Wiener process (Tuckwell, 1988):

(A 2)

The latencies that result from such a process follow an inverse Gaussian distribution such that:

(A 3)

It is therefore possible to exactly match the noise of our integrate and fire model to the noise of the Poisson model such that Eqn 12 is satisfied. This, however, means that:

(A 4)

and therefore,

(A 5)

We note that since *r*(*v*)δ*t*=1 for all practical purposes, it follows that CV(δ*N*)≫1, which effectively means that this model is no longer history dependent, but rather dominated by instantaneous input (see Discussion). In fact, an implementation of this model behaves almost exactly like the Poisson model (data not shown).

To keep the model history dependent and the comparison meaningful, we therefore introduced noise that approximates the noise in the Poisson model without leading to a coefficient of variation equal to one. We note that our noise ε, which we add in each time step affects the value *N*(*t*) of our integrator rather than changing the latency *L* directly. We call this error *E_N_*. After *n* time steps, *E_N_* will follow the relation:

(A 6)

From the diagram in Fig. A1 together with Eqn 7, we see that  is related to *S* by *r*(*v*), the slope of our integrator. In general  can be estimated based on the spread between the points:

For , *P*_2_ is not defined and we note that . We use the location of this point to set *var*(*E_N_*) as illustrated in Fig. A1:

(A 7)

Based on Eqns A6 and A1 our error ε per time-step needs to be distributed as follows:

(A 8)

We note that equating *n*σ^2^ to 0.5^2^ results in a total error that is indeed smaller than  as desired to keep our model history dependent. However, the introduction of Gaussian noise into our integrator has the desirable property that the resulting latencies *L* are not symmetrically distributed around the mean  but rather have a right-tailed distribution qualitatively similar to the exponential distribution of latencies obtained with the Poisson model (also see Fig. 2). We also note that random walk models with drift, such as our noisy integrate and fire model, have been used to describe stochastic processes in neuronal firing (Gerstein and Mandelbrot, 1964; Shadlen and Newsome, 1994). When presenting our models with gratings of varying speed we adjust σ at each time step such that:

(A 9)

#### Generalization to the leaky integrator

For our leaky integrator model, we make the same assumptions about noise with *E_N_* distributed as in Eqn A7. Our goal is therefore to estimate σ such that Eqn A7 holds. We note that for the leaky integrator with leak μ at each time step *i*:

(A 10)

Note that if

(A 11)

(A 12)

Together with Eqn A7:

(A 13)

### Model of the retina as an edge counter

In this section, we present a simple model of the retina as an edge counter. The model does not attempt to be quantitative, the fact that in this model the retina is a semi-circumference as opposed to a semisphere is already unrealistic. Nevertheless, it aims to be qualitative or heuristic and suggests a way in which input to the models presented in the main text of the paper, namely the rate *r*(*v*) in the integrate and fire model or the Poisson rate λ(*v*), may arise.

Consider the situation depicted in Fig. 4A: a retina *R* of radius *r* looking down on a screen *S* on which a square wave grating is presented. The question we wish to address is: what would be the output at any instant as a measure of the rate at which edges were passing by below it on the screen? In order to make our model more general we will consider the dependence of this activity as a function of both the speed of the grating and its spatial frequency. The dependence of latency on these two parameters was tested for 26 fish and is shown in Fig. 4B.

The angle θ is related to the coordinate *x* on the screen *S* by:

(A 14)

Edge density on *S* is constant (independent of *x*). Assume a grating of spatial width *w*=*f*^−1^ such that there are *f* periods per mm. This implies that we have *xf* periods in the interval [0, *x*] on *S*, which implies *xf* periods in the angular interval [0, θ], which, in turn, corresponds to *sf* tan θ periods in that same interval [0, θ]. Differentiating this with respect to θ, we obtain the period density per radian ρ_period_:

(A 15)

By inverting Eqn A15 we obtain the angular extent of a full period as a function of the angle θ. We will assume that: (1) detectors are numerous, of equal size *d* and that they tile the retina uniformly; (2) a detector can count at most one passing edge at a time; if two or more edges pass its receptive field simultaneously the contribution of the detector is zero; and (3) an edge must spend a minimum amount of time Δ*t* over the detector's receptive field in order to be counted.

In order to take into account point 2 above, we can calculate that there is a whole period in a detector placed at an angle θ when the detector's angular extent *d*/*r* is equal to the angular extent of a whole period. This is the case for angles which exceed θ^lim^, which is given by:

(A 16)

where we have inverted Eqn A15 with ρ=*r/d*.

We now take into account point 3 above. From Eqn A14 we can obtain a relation for the angular velocity dθ/d*t* of the grating on the retina

(A 17)

The angle advanced in a time Δ*t* is

(A 18)

but according to point 3, this must be less than the angular extent *d*/*r* of a receptor, from which we obtain:

(A 19)

Now consider a grating moving with constant speed *v* mm s^−1^ on *S*. Within any interval of size *I*≪*f*^−1^ on *S*, *vf* edges pass it per second. Given our assumptions and as calculated above, this is true for all the inverse images on *S* of the detectors for angles θ such that θ_min_≤|θ|≤θ^lim^. The activity per second *A* is therefore given by:

(A 20)

If this activity is integrated following a non-leaky integrator, which fired when the total activity reached a threshold *T*_th_, this would give a latency *L* given by:

(A 21)

such that

(A 22)

This is shown in Fig. 4C. We note that the upper and lower limits in the integral (Eqn A20) arise as geometric constraints in our model (namely points 2 and 3) above, and that they are responsible for the interesting behavior we observe in Fig. 4C: the deviation from linearity and the appearance of a long latency region for a grating of short spatial period.

We find that, despite the numerous assumptions and shortcomings of the model, it still manages to capture the most general features of period and velocity dependencies of the data shown in Fig. 4B: the increase in latency as spatial period increases, in particular for slow velocities, and the strip of long latencies for very fast and thin gratings. We conclude that it is definitely possible for the brain to have a mechanism by which it may compute a rate which can be used as input for either the integrate and fire model or the Poisson model we have considered in the main text, and that this rate may be calculated even by integrating over the retinal outputs.
